# Cognitive biases: potential behavioral marker for future development of postpartum depression and childbirth-related post-traumatic stress disorder

**DOI:** 10.3389/fpsyt.2025.1650453

**Published:** 2025-08-20

**Authors:** Vanessa Cywiak, Ido Solt, Nur Givon-Benjio, Eyal Fruchter, Hadas Okon-Singer

**Affiliations:** ^1^ Department of Obstetrics and Gynecology, Technion, Israel Institute of Technology, Haifa, Israel; ^2^ Department of Psychiatry, Technion- Israel Institute of Technology, Haifa, Israel; ^3^ School of Psychological Sciences, University of Haifa, Haifa, Israel; ^4^ The Integrated Brain and Behavior Research Center (IBBRC), University of Haifa, Haifa, Israel; ^5^ Faculty of Medicine, Technion, Israel Institute of Technology, Haifa, Israel; ^6^ Division of Maternal-Fetal Medicine, Department of Obstetrics and Gynecology, Rambam Medical Center, Haifa, Israel; ^7^ Research Unit, Psychiatric Unit, Rambam Medical Center, Haifa, Israel; ^8^ Max Planck Institute of Human Cognitive and Brain Sciences, Leipzig, Germany

**Keywords:** postpartum depression, postnatal depression, childbirth post-traumatic stress disorder, cognitive biases, preventative care, cognitive training, attention bias, interpretation bias

## Abstract

Postpartum Depression (PPD) and Childbirth Post-Traumatic Stress Disorder (CB-PTSD) are psychiatric conditions that cause significant distress. Yet despite their high prevalence and decades of research, knowledge about causal cognitive mechanisms that may assist in predicting or preventing these conditions is still missing. One characteristic of PPD and CB-PTSD that may contribute to their early prevention is the existence of cognitive biases concerning future parenting. Cognitive biases have been shown to play an important role in the etiology of various other psychiatric disorders, including depression and PTSD, suggesting they might have a similar role in PPD and CB-PTSD. From a theoretical perspective, understanding the associations between cognitive biases, PPD, and CB-PTSD may lead to novel theoretical models and research avenues. Additionally, understanding the cognitive mechanisms underlying PPD and CB-PTSD has several important clinical implications, such as *early detection*, *preventative care*, and developing *individually tailored cognitive therapies* focusing on these specific biases.

## Introduction

Approximately 10–20% of women experience Postpartum Depression (PPD)* or Childbirth-Related Post-Traumatic Stress Disorder (CB-PTSD) after giving birth* (1, 2). Both conditions are associated with significant psychological, health, and familial challenges (3, 4) including child neglect and, in extreme cases, maternal suicide or infanticide (4–6). Women with high-risk pregnancies are more vulnerable (7, 8). Moreover, 8% to 25% of fathers or partners also experience PPD symptoms (9). Beyond the individual suffering of mothers^1^ ^2^ ^3^+; and partners, PPD and CB-PTSD are associated with problems in attachment between mothers and their newborns (10, 11). Children of mothers with PPD are also more likely to exhibit psychological and cognitive-emotional difficulties during adolescence (12).

This paper proposes that cognitive biases may serve as early markers, or warning signs, for the potential development of PPD and CB-PTSD. Identifying such markers could contribute to the prevention of these disorders. To support this proposal, the paper provides a brief review of relevant literature, explores the role of cognitive biases as early indicators, and outlines directions for future research.

## Postpartum depression and CB-PTSD: comorbidity, prevalence, and symptoms

PPD is categorized as a mood disorder and a subtype of major depressive disorder (MDD), with symptoms typically emerging within the first four weeks after childbirth (1, 13). Common symptoms include depressed mood, fatigue, anhedonia, low self-esteem, difficulty concentrating, loneliness, irritability, anxiety, feelings of guilt, hopelessness, and reduced self-confidence (14). The prevalence of PPD ranges from 10% to 15% in the general postpartum population (6, 15) and rises to 27%–44% among women with high-risk pregnancies (8). A history of depression is one of the most robust predictors of PPD (16). Other risk factors include low social support (17), socioeconomic disadvantage, unplanned pregnancy (1), poor sleep quality (18), perceived low quality of life (19), older maternal age, low relationship satisfaction (20), excessive worry about motherhood during pregnancy (21), acute pain following childbirth (22), self-reported pain during pregnancy (23) and “maternity blues”, a temporary condition experienced during the first days postpartum, may increase the likelihood of developing PPD (24, 25).

Several biological mechanisms have been implicated in the development of PPD. Lower levels of salivary cortisol have been observed in mothers, fathers, and infants within families affected by PPD (26). Mothers with PPD are more likely to exhibit lower levels of the GG genotype versus the A-carrying variant of the oxytocin receptor gene, a hormone associated with affective touch and lower empathic engagement with their babies (27). Gonadal steroid fluctuations also appear to play a significant role in the onset of postpartum mood disturbances (28).

Another condition during the postpartum period is CB-PTSD, classified within the category of trauma- and stress-related disorders, and follows the same diagnostic criteria as PTSD, when the traumatic event must be the childbirth experience (1, 13). According to DSM-V (13), PTSD symptoms include intrusive re-experiencing of the event, avoidance of reminders or places associated with the trauma, and heightened physiological arousal (29). In the context of CB-PTSD, affected individuals may experience distressing and intrusive memories or nightmares about childbirth, irritability, hypervigilance, sleep disturbances, difficulty concentrating, emotional withdrawal, and feelings of guilt. They may also avoid discussing or thinking about the labor or birth experience (30–32).

The prevalence of CB-PTSD in community samples ranges from 1% to 6% (33), with significantly higher rates among women who experienced high-risk pregnancies, 9.9% for those with clinically significant symptoms, and up to 11.9% for those with subclinical symptoms (34). Several psychological and social risk factors have been identified, including perceiving the birth experience as traumatic or negative, intense fears for one’s own health or the baby’s health during labor (2), and a history of childhood sexual abuse or domestic violence (1).

Additional physiological and obstetric variables have been linked to the development of CB-PTSD. These include prolonged labor (lasting more than six hours), postpartum hemorrhage (35), and severe perineal tearing (36). Women who experienced hyperemesis gravidarum reported significantly higher CB-PTSD symptom levels compared to those with either no or milder forms of pregnancy-related nausea, with effects lasting up to two years postpartum (37). Despite this evidence, results are mixed, and other studies found no significant associations between CB-PTSD and obstetric factors such as type or duration of labor, severity of hemorrhage, low APGAR scores, or breastfeeding challenges (38).

Research consistently demonstrates a strong relationship between PPD and CB-PTSD, including shared risk factors and high comorbidity (1, 39–41). Evidence further shows that PPD and CB-PTSD are associated with postpartum anxiety (15, 42, 43) and with maternal bonding and attachment (44).

## Preventive treatment of postpartum depression and CB-PTSD

Similar to other psychiatric disorders, there is growing medical and scientific interest in shifting toward preventive care and early detection of PPD and CB-PTSD, particularly by identifying behavioral and physiological markers (45, 46). Currently, both conditions are often diagnosed only after symptoms have become pronounced. Although maternal depression screening is recommended during routine pediatric care, many women underreport symptoms during pediatric visits (47). This underreporting can result in missed referrals, especially when relying on current screening thresholds (47). The delay in diagnosis is often influenced by sociocultural pressures, such as the desire to appear as a “good” mother, internalized stigma around mental health (47), guilt or shame about not feeling joy after childbirth, and societal expectations of motherhood (40).

Compounding this issue, research has identified multiple sociodemographic, physiological, psychological, environmental, and pregnancy-related risk factors for PPD and CB-PTSD (48, 49), such as older age, lack of sleep, psychiatric history, high anxiety, and lack of social support (1, 18, 20, 39, 40). While each self-report survey may be a quick and cost-effective screening tool, the implementation of these questionnaires lacks standardized protocols. Comprehensive screening requires multiple questionnaires, adequate clinical training, and structured referral pathways, elements frequently missing in routine care (47, 48, 50, 51). Moreover, CB-PTSD is often overlooked during postpartum screening, due to its symptom overlap with PPD and its lower public and clinical visibility (39). To address these gaps, researchers recommend the development and validation of comprehensive prenatal screening tools that capture a broader range of risk factors, while also improving objectivity and medical relevance (47, 48, 51).

These obstacles underscore the urgency of identifying early behavioral markers that can support timely and targeted interventions. In this context, cognitive biases hold promise as early indicators, not only to enhance early detection, but also to guide preventive cognitive interventions in pregnant individuals at increased risk for PPD and CB-PTSD.

## Cognitive biases associated with postpartum depression and CB-PTSD

Cognitive biases refer to systematic distortions in the processing of emotional information. These can include biased attention toward threatening stimuli, distorted interpretation of ambiguous situations, altered memory for negative events, or negative expectations about the future (52, 53). Such biases are known to contribute to the onset and maintenance of various psychiatric conditions (54), including depression (55, 56), anxiety disorders (56, 57), phobias (58, 59), PTSD (60, 61), and eating disorders (62).

Accumulating research demonstrates that specific cognitive biases characterize PPD and CB-PTSD (**Table 1**). For example, mothers with PPD show greater attention bias toward images of infants compared to adults (63–65). Depressed pregnant women also disengage more quickly from distressed infant faces than from non-distressed ones, a pattern not observed in non-depressed women (64, 66). Notably, this tendency to stronger attentional bias towards distressed infant faces was associated with more positive mother–infant bonding reported after birth (66).

**Table 1:** Summary of papers described in this article on cognitive biases and processes.

Author, year, and country.	Tasks	Population	Time of assessment	Main finding
Gil et al. (2011), France (63).	Emotion recognition of infants' and adults' faces: -The emotional facial expression task	Postpartum: Sub-clinical PPD and healthy women	Postpartum: 3 days after delivery.	1. Women with higher depressive symptoms tended to interpret neutral infant faces as sad, but their perception of other infant emotions was unaffected. 2. The intensity of depressive mood did not influence how emotional expressions in adult faces were judged. 3. Greater anxiety was linked to rating neutral infant faces as less neutral and sadder, and to judging angry infant faces as more disgusted. 4. Higher anxiety also led to perceiving neutral and angry adult faces as showing more disgust.
Flanagan et al. (2011), USA. (65)	Emotion recognition in adults. 1. Ekman 60 Faces Test: recognizing prototypical facial expressions. 2. Emotion Hexagon Task	Postpartum: PPD MDD Healthy non-mothers.	Up to one month after childbirth	1. Women with PPD showed significantly poorer recognition of fear, happiness, and disgust across both tasks, and of anger in one task, compared to controls. 2. Compared to women with MDD, the PPD group was less accurate in identifying anger and disgust expressions. 3. In contrast, MDD participants struggled more than those with PPD in recognizing happy faces.
Pearson et al. (2010), UK (64).	Attention and inhibition bias measures of Infants' and adults' emotional pictures: -Go/No-Go task.	Prepartum: with and without depressive symptoms.	Up to 12 weeks of pregnancy	1. Non-depressed women showed an attentional bias toward distressed infant faces, demonstrated by slower disengagement compared to non-distressed infant faces. 2. Women with depressive symptoms did not show this bias; instead, they tended to disengage more quickly from distressed infant faces than from non-distressed ones (a trend toward a reverse effect). 3. Depressive symptoms did not affect overall attention toward infant vs. adult faces, nor did they influence attention to emotional expressions in adult faces (negative, neutral, or positive).
Pearson et al. (2011), UK (66).	Attention and inhibition bias measures of infants’ emotional pictures: -Go/No-Go task, prepartum depression, and bonding with the newborn after delivery.	Prepartum: with and without depressive symptoms.	Late pregnancy (range 34–39 weeks).	1. During late pregnancy, women exhibited an attentional bias toward distressed infant faces. 2. Stronger attentional bias towards distressed infant faces was associated with more positive mother–infant bonding reported after birth. 3. Women who reported stronger postpartum bonding showed distinct patterns of processing infant distress even before childbirth.
Webb & Ayers (2015), UK (67).	Systematic review for cognitive biases and peripartum, related to PPD and CB-PTSD.	Not apply	Not apply	1. Emotional biases in perinatal women with depression or anxiety are specific to infant emotions, with greater sensitivity to sad infant faces and reduced sensitivity to happy ones. These biases are less evident when adult faces are used. 2. Mothers with depressive or negative affect show perceptual biases, tending to interpret infant facial expressions as more negative than controls. 3. Women with anxiety or depression, during pregnancy or postpartum, have difficulty identifying positive infant emotions, but may be more accurate in recognizing negative ones. 4. Faster disengagement from infant emotional expressions was observed in pregnant women with depressive symptoms, possibly reflecting reduced emotional engagement. 5. These emotional processing biases were found as related to lower maternal sensitivity in mother–infant interactions
Arteche et al. (2011), USA (69).	Emotion recognition in infants through the identification of babies' facial emotions.	Postpartum: PPD, GAD, Healthy non-mothers.	Postpartum: between 10 and 18 months after delivery	1. Mothers with postnatal depression did not differ from controls in their recognition of sad infant faces. 2. Mothers with GAD identified happy infant faces at a lower intensity than controls, and a similar trend was observed in mothers with depression. 3. Overall, mothers of young children were more sensitive to happy than sad infant faces. However, those with PPD were less accurate in identifying happy faces than controls. 4. Both PPD and GAD groups showed reduced accuracy in recognizing happy faces, suggesting that this difficulty may reflect a broader emotional processing issue, not specific to depression. 5. The study found no significant cognitive bias differences between PPD and GAD groups in recognizing or reacting to infant faces, indicating similar patterns of impairment across both conditions.
Bloch (2022), Israel (28)	Emotional cognitive measures through: 1. Self−referential encoding and incidental recall task (SRET). 2. Dot−probe task (DPT). 3. Emotional Stroop.	Postpartum: with and without history of PPD.	Postpartum: at least one year after delivery. Two assessments: once during the follicular phase (days 10-12) and once during the luteal phase (days 24-26).	1. Women with a history of PPD (hPPD) reported more negative self-perceptions during the luteal phase compared to the follicular phase, while women without PPD history showed a non-significant trend in the opposite direction. 2. In the luteal phase, hPPD women showed a negative cognitive bias: they endorsed more negative traits, remembered more negative and fewer positive self-descriptions (SRET), and paid more attention to emotional than neutral stimuli (E-Stroop). 3. These cognitive biases were not accompanied by mood changes, suggesting they reflect a trait-like processing vulnerability, not just mood reactivity. 4. No phase-specific attentional bias was found on the dot-probe task, indicating that attentional effects may vary by task type.
Bjertrup et al. (2021), Denmark (68).	Emotion recognition and emotion intensity ratings of infant faces. 1. The facial expression recognition task (FERT) 2. Ratings of Emotional Infant Stimuli 3. The Screen for Cognitive Impairment in Psychiatry (SCIP)	Prepartum: MDD, Bipolar Depression, Healthy Pregnant, Healthy non-pregnant women. Postpartum: Sub-clinical PPD symptoms	Prepartum: 27-40 weeks of pregnancy Postpartum 1: 2–25 days after birth. Postpartum 2: 10–36 months after birth.	1. Pregnant women with unipolar depression, even in remission, rated infant cries more negatively than healthy pregnant women. This negative bias was independent of medication use. 2. Pregnant women with bipolar disorder rated infant happy faces more positively than healthy pregnant women, and showed greater recognition of positive vs. sad adult faces, but did not differ in their ratings of infant cries. 3. Across all groups, infant cry ratings were the strongest predictor of PPD, even when controlling for subclinical depressive symptoms during pregnancy. 4. Pregnancy itself was associated with less negative responses to infant cries and slightly reduced cognitive performance, but no evidence of a threat bias.
Bjertrup et al. (2023), Denmark (72)	Emotion recognition of infants' sounds. 1. The infant sounds - Oxford Vocal (OxVoc) sounds database 2. two video clips	Pregnant: Healthy Postpartum: PPD and Healthy	Prepartum: second and third trimesters of pregnancy.	1. Greater negative emotional responses to a distress video during pregnancy were linked to higher levels of depressive symptoms (EPDS scores) within the first 2 months postpartum. 2. Stronger negative interpretations of distressed infant cries also predicted higher EPDS scores and increased risk of PPD within 6 months postpartum. 3. These associations remained significant even after adjusting for prenatal depressive symptoms, prior depression history, income, financial stress, and childhood trauma. 4. Emotional reactivity to the video had higher predictive validity (better sensitivity and specificity) for PPD than reactions to the infant cry sounds.
Edvinsson et al. (2017), Sweden (71).	Emotional attention bias in pregnancy: -The emotional Stroop task, presenting emotionally charged delivery-related words	Prepartum: MDD (with and without comorbid anxiety) Healthy Postpartum: PPD (with and without comorbid anxiety) Healthy	Prepartum: pregnancy weeks 35–39	1. Postpartum depression was associated with faster responses to both positive and negative emotional words compared to nondepressed women. 2. Higher depression scores (MADRS/EPDS) were linked to reduced emotional interference during the task. 3. Emotional content effect: Participants showed slower reaction times to negative obstetric words and faster responses to positive words. 4. Perinatal status (e.g., pregnant vs. postpartum) had no significant effect on attention bias or its interaction with emotion.
Dale-Hewitt et al. (2012), UK (73).	Emotional attention bias in postpartum women with CB-PTSD symptoms: -The emotional Stroop task, presenting emotionally charged delivery-related words	Postpartum: women with CB-PTSD symptoms (PTSD-Q and IES).	Postpartum: Between 1.5 and 6 months after delivery.	1. Higher PTSD symptoms, measuring mainly the presence of the avoidance and intrusion dimensions of PTSD, were associated with faster reaction times to labor-related words, indicating an attentional bias. 2. A more negative labor and delivery experience was linked to faster responses to labor words, also reflecting an attentional bias. 3. Depressive symptoms were not associated with attentional bias to labor-related content. 4. There was no significant difference in reaction times between labor words and labor-control words, suggesting limited stimulus-specific effects for the PTSD-Q measure.
Hampson et al. (2015), Canada (86).	Working memory (WM) tasks related to hormones: Neuropsychological tests: -Spatial working memory -Self-Ordered Pointing -Paragraph Recall -Corsi Blocks -Mooney–Harshman Closure Six hormones: (progesterone, estriol, estradiol, testosterone, cortisol, dehydroepiandrosterone)	Prepartum: Pregnant (third trimester) and non-pregnant women. Postpartum: two groups of pregnant women and non-pregnant women, within the 4 months after delivery. With and without MDD or PPD.	Pregnant women: the first visit between 34 and 38 weeks of gestation and the second postpartum, 3 to 4 months later. Postpartum control group: the first visit 4 to 12 weeks after delivery, and the second visit 3 to 4 months later.	1. WM was impaired in pregnant women with clinical depression (Preg+), but not in healthy pregnant women (Preg−) or non-pregnant controls. 2. The WM deficits were specific to depression during pregnancy, as explicit memory remained unaffected and WM performance returned to normal postpartum when mood improved. 3. Healthy pregnant women (Preg−) demonstrated high WM performance, even matching or exceeding that of non-pregnant controls, suggesting that WM decline is not typical of healthy pregnancies. 4. WM deficits in Preg+ women were reversible, reinforcing previous research on gestational memory impairment (GMI) and its association with mood state. 5. Higher estradiol levels were associated with fewer WM errors, while other hormones (progesterone, estriol, DHEA, testosterone, cortisol) did not differ significantly between groups or predict WM performance. 6. Only estradiol levels and depression severity (MADRS scores) were significant predictors of WM performance, while cortisol and sleep quality had no predictive value.
Pio de Almeida et al. (2012), Brazil (17).	Recall and working memory tasks in postpartum women and men. Neuropsychological test: word span test, to evaluate: -Working (immediate) -Short-term (recalled) memories.	Postpartum: Women and men (parents).	Does not specify: live births, born from March to December 2008.	1. PPD was significantly associated with impaired immediate and recall memory for neutral and negative stimuli, even after adjusting for medication use and alcohol consumption 2. Working memory scores were lower in individuals with PPD compared to those without. 3. Positive memory (immediate or recall) showed no significant impairment, though a marginal p-value suggests a possible trend. 4. Of the socioeconomic factors assessed, only sex was linked to higher PPD risk, but it did not affect memory performance.
Messinis et al. (2010), Greece (87).	Multiple cognitive tasks to evaluate: -Cognitive attention and psychomotor speed. -Working memory (visual and auditory). -Recall, interference, and delay recall auditory memory. -Controlled Search Speed. -Executive functioning. Neuropsychological test: -Verbal fluency test composed of phonological and semantic cues. -The Rey Auditory Verbal Learning Test (RAVLT). -Trail Making Test, parts A and B (TMT). -Ruff 2 and 7 Selective Attention Test.	Postpartum: women after delivery. With and without PPD, and a non-pregnant healthy women group.	Postpartum: between one and two months after delivery.	1. Postpartum women performed worse than healthy controls on the initial verbal learning trial and the interference trial (List B) of the RAVLT, but no differences were found across other trials or between the two postpartum subgroups. 2. Postpartum women with depression showed impaired performance on the Trail Making Test Part B, even after adjusting for psychomotor speed, indicating deficits in cognitive flexibility and executive functioning. 3. Verbal fluency and visual attention tasks showed no significant differences across groups, suggesting these domains remain intact regardless of postpartum or mood status.

GAD, general anxiety disorder; PPD, postpartum depression; CB-PTSD, child-birth-related posttraumatic stress disorder; PTSD, posttraumatic stress disorder; WM, working memory; MADRS, Montgomery-Åsberg Depression Rating Scale; MDD, major depressive disorder; EPDS, Edinburgh Postnatal Depression Scale; IES, impact of events scale; PTSD-Q, Posttraumatic Stress Disorder Questionnaire.

The table includes only studies that focus on computerized tasks designed to measure cognitive biases.

In terms of interpretation bias, the tendency is not yet clear. Mothers with PPD tend to rate neutral infant faces as sad (63) and are more sensitive to negative facial expressions in infants compared to happy ones (63, 67, 68). While Gil et al. [2011 (63)] found no significant impact of depressive symptoms on the recognition of positive emotions (e.g., smiling infants) among mothers with PPD three days after delivery, Arteche et al. [2011 (69)] reported that women with PPD more than 10 months postpartum did not differ from nonpregnant women in the recognition of sad infant faces, and were less accurate in identifying expressions of happiness in infant faces. The same tendencies were reported for postpartum women with anxiety disorder (see (67) for more information, or **Table 1** in this manuscript).

Research suggests that women with a history of PPD may continue to exhibit cognitive vulnerabilities beyond the postpartum period, particularly during the luteal phase of the menstrual cycle (28). During this phase, they tend to show more negative self-evaluations and display memory and attentional biases toward negative information, even when not currently experiencing depressive or mood symptoms (28). As noted above, a history of MDD (16, 70) or bipolar disorder (68) increases the risk of developing PPD. PPD can also emerge in women without a prior psychiatric history (40); however, different cognitive bias profiles have been observed in women with and without a prior history of MDD or bipolar disorder (68, 71).

Finally, reduced accuracy in recognizing happy infant faces may signal impaired attunement to a newborn’s needs. This reduced responsiveness may negatively affect early mother-infant bonding, contributing to relational difficulties and potentially hindering the child’s emotional development (66, 67, 69).

## Can cognitive biases predict future development of postpartum depression and CB-PTSD?

Despite the importance of identifying cognitive biases in perinatal mental health, only a few studies have investigated biases during pregnancy and related them to the development of postpartum disorders. One study found that stronger negative reactions to a crying infant during pregnancy significantly predicted later development of PPD, even after controlling for subclinical depressive symptoms (68). Another study used an emotional Stroop task and showed that depressed pregnant women undergoing treatment exhibited greater emotional interference from negatively valenced obstetric words (e.g., “pain,” “vacuum extraction,” “brain injury”) than either untreated depressed women or healthy pregnant controls (71). Notably, participants who later developed PPD showed faster reaction times to both positive and negative obstetric words, suggesting a generalized attentional bias to emotional obstetric information.

Recent findings further support the predictive value of interpretation biases. When asked to assess the emotional states of infants in short videos and audio clips, pregnant women who interpreted infant distress more negatively were more likely to develop depressive symptoms within the first two months postpartum (72). Moreover, harsher evaluations of the most distressing infant cries were particularly predictive of higher depressive symptom levels, but the emotional reactivity to the video had higher predictive validity. These results suggest that even adjusting for prenatal depressive symptoms, prior depression history, income, financial stress, and childhood trauma, the association of pregnant women with PPD is still stronger in this study, and negative interpretation biases may be at elevated risk for developing PPD (68, 72).

Research investigating the predictive utility of cognitive biases for CB-PTSD remains limited compared to that for PPD. Much of the available literature focuses on women with prior PTSD or those who experienced trauma during pregnancy, rather than on trauma stemming specifically from the childbirth experience. Nevertheless, a few studies provide important insights. One study used an implicit, computerized Stroop task to assess attention bias in women with subclinical symptoms of CB-PTSD. Attention bias away from labor-related words was associated with higher post-traumatic stress and more negative childbirth experiences (73). Additional research explored more explicit, reflective measures related to memory and narrative processing. For example, studies have examined how writing about the childbirth experience, memory distortions of labor pain, and cultural perceptions of unplanned or traumatic births may influence the risk of developing CB-PTSD (74–78).

These studies provide preliminary evidence that cognitive biases may serve as early indicators of vulnerability to PPD and CB-PTSD. By examining both correlational and causal links between specific biases and postpartum psychopathology, *research can begin to define cognitive profiles for individuals at elevated risk*. Recent developments in computational psychiatry further support this approach. Studies using machine learning (ML) and nonlinear analytic methods have successfully predicted symptoms of anxiety and depression based on cognitive bias patterns (52, 79, 80), as an addition to the traditional analysis. Recent studies demonstrate the potential of ML to improve predictive accuracy. For example, a natural language processing (NLP) model analyzing women’s childbirth narratives achieved 85% sensitivity and 75% specificity in identifying CB-PTSD symptoms (81). Similarly, a population-based study applying Random Forest models to predict PPD reported an AUC of 0.884, indicating high diagnostic accuracy (82), with implied sensitivity and specificity often ranging between 70–90% and 60–85%, depending on the dataset and thresholds used.

These advances highlight the potential to identify and differentiate several variables specifically related to PPD and CB-PTSD, revealing a complex relationship in the data without assuming the relationships (predictor or predicted) (48), and enabling more accurate predictions for the population through actual or retrospective data (83).

## Cognitive control difficulties and their relationship with postpartum depression and CB-PTSD

Cognitive biases are commonly associated with reduced cognitive control over emotional processing and reactions (54). Cognitive control is essential for flexible, goal-directed behavior, particularly in uncertain situations. It enables individuals to override automatic responses, inhibit irrelevant information, shift attention, and update working memory, skills that help allocate mental resources and prioritize task-relevant information (84, 85).

In the context of PPD and CB-PTSD, evidence suggests impairments in executive functions. For instance, Hampson et al. (86) demonstrated working memory decline in pregnant women with depressive symptoms; Pio de Almeida et al. (14) reported deficits in working and short-term memory in depressed parents; and Messinis et al. (87) found that women with postnatal depression performed worse on tasks involving learning and attention-switching compared to controls. Despite these findings, research on cognitive control in postpartum psychopathology remains limited and warrants further exploration.

Some studies have investigated interventions targeting these cognitive processes. Di Blasio et al. (41) showed that writing about the childbirth experience significantly reduced PPD symptoms 96 hours after delivery, with further reductions in both PPD and CB-PTSD, observed following a similar intervention three months postpartum.

DeJong et al. (85) proposed a cognitive control model linking PPD, rumination, parenting, and child development. They suggest that women with PPD tend to focus on negative information, which then shapes ruminative thinking. Supporting this, Denis and Luminet (88) found that rumination predicted PPD symptoms and maternal self-esteem at one month postpartum, while neuroticism predicted outcomes at one year.

Ford et al. (89) adapted a general PTSD cognitive model to CB-PTSD, including social support as a potential predictor. Their model explained 23% of the variance in CB-PTSD symptoms at three weeks postpartum, with no improved prediction when social support was added to the model. However, at three months postpartum, social support, partially mediated by post-traumatic cognitions, improved the model’s predictive power (16% variance explained), suggesting that social support becomes more critical over time. These findings affirm that CB-PTSD over time is predicted by the influence of early social support.

Peñacoba et al. (90) examined cognitive variables linked to PPD were positively associated with neuroticism and negatively associated with extroversion. Worries and an external locus of control influenced both anxiety and PPD, whereas expectations about childbirth affected only anxiety. Additionally, anxiety during pregnancy emerged as a strong independent predictor of PPD.

Optimism was associated with fewer depressive symptoms during pregnancy and at two weeks postpartum, while self-esteem consistently predicted lower depression levels across the six months. Self-esteem may serve as a more stable protective factor against early postpartum depression (91, 92). Similarly, women with poor or moderate coping strategies were more likely to report symptoms of CB-PTSD compared to those with effective coping mechanisms (93). Cultural context also shapes coping behaviors such as accepted social support, emotion-focused coping, or religious coping. Importantly, higher self-esteem among postpartum women was linked to increased use of positive coping strategies (51, 92). Moreover, women who demonstrated higher levels of positive coping during the third trimester were less likely to develop postpartum depression (51).

Collectively, these models underscore the potential role of executive control with the effects of the personality traits in PPD and CB-PTSD. However, more research is required to reach firm conclusions and establish gold-standard theoretical models.

## Summary and future directions

Accumulating evidence proposes that cognitive biases may serve as behavioral markers for early diagnosis and prevention of PPD and CB-PTSD. Few studies have explored attentional biases across genders (9, 94). None have examined differences between biological and non-biological caregiver parents.

Systematic studies comparing cognitive biases in large cohorts of pregnant women are scarce. Additionally, existing research often focuses on isolated cognitive biases or risk factors, neglecting how different factors might interact. The combined cognitive bias hypothesis (95), originally proposed in the context of depression, suggests that biases interact. Applying this integrated perspective to PPD and CB-PTSD may clarify their underlying mechanisms. Supporting this, negative interpretation bias predicted higher levels of fear of childbirth, whereas attention and memory biases showed no significant associations (96).

Cognitive bias measures may offer an implicit and objective alternative to traditional self-report tools, which are prone to subjective biases (97, 98). These tasks are low-cost, easy to administer, and require no special equipment, making them accessible and practical for early screening and preventive care globally. When used alongside self-reports, they may improve diagnostic accuracy. Emerging evidence also suggests that cognitive interventions, such as attention bias modification and cognitive bias modification (99, 100), could alleviate symptoms of PPD and CB-PTSD by targeting maladaptive thought patterns (biased attention-orienting or negative interpretations). These interventions leverage neuroplasticity to improve emotional and cognitive outcomes and provide a direct method for testing the causal role of cognitive mechanisms in symptom change (101).

Although mixed findings exist [for discussion, see (102)]. Cognitive training has shown effectiveness in reducing symptoms of depression (56, 103), anxiety (100), social anxiety (104), PTSD (105), rumination (106), and schizophrenia (107). However, its use in PPD and CB-PTSD remains underexplored. Hirsch et al. (108) conducted the first training with pregnant women (>16 weeks gestation) reporting high worry. Their cognitive bias modification for interpretation training promoted positive interpretations of ambiguous scenarios, reduced negative thought intrusions, and induced a more positive interpretation bias, supporting a causal link between interpretation bias and worry during pregnancy.

## Conclusions

Cognitive biases may play a key role in the development and maintenance of PPD and CB-PTSD. They may function as early warning signs for their development, offering potential avenues for early identification and prevention. Growing evidence indicates that distinct cognitive biases are characteristic of both disorders. Future research should expand on this evidence, focusing on diverse populations, integrated bias models, and longitudinal designs to refine theoretical understanding and enhance clinical practice.

## Data Availability

The raw data supporting the conclusions of this article will be made available by the authors, without undue reservation.

## Footnotes

1 In this paper, we refer to the term women because currently, the research relevant to the topic under discussion has only been conducted on women.

2 *This paper uses the terms postpartum depression (‘PPD’) and postnatal depression (‘PND’) interchangeably. Both terms refer to depression that is delivery-related, in line with recent literature. The same is true for the use of postpartum post-traumatic stress disorder (‘PP-PTSD’) and childbirth PTSD (‘CB-PTSD’).

3 +;In this paper, the term 'mother' refers to the partner or caregiver who predominantly fulfills the traditional maternal role.
